# Di-μ-acetato-κ^3^ *O*,*O*′:*O*;κ^3^ *O*:*O*,*O*′-bis­[(acetato-κ^2^ *O*,*O*′)(1,10-phenan­throline-κ^2^ *N*,*N*′)cadmium(II)]

**DOI:** 10.1107/S1600536808029462

**Published:** 2008-10-22

**Authors:** Miguel Angel Harvey, Sergio Baggio, María Teresa Garland, Ricardo Baggio

**Affiliations:** a Universidad Nacional de la Patagonia, Sede Trelew, 9100 Trelew, Chubut, Argentina; b CenPat, CONICET, 9120 Puerto Madryn, Chubut, Argentina; c Universidad Nacional de la Patagonia, Sede Puerto Madryn, Argentina; d Departamento de Física, Facultad de Ciencias Físicas y Matemáticas, Universidad de Chile and CIMAT, Casilla 487-3, Santiago de Chile, Chile; e Departamento de Física, Comisión Nacional de Energía Atómica, Buenos Aires, Argentina

## Abstract

The title compound, [Cd_2_(C_2_H_3_O_2_)_4_(C_12_H_8_N_2_)_2_], consists of dimeric units built up around a crystallographic symmetry centre. Each cadmium(II) unit is chelated by a 1,10-phenanthroline (phen) group and two acetate ligands, one of which also acts as a bridge, linking both seven-coordinated cadmium(II) centres. The crystal structure is governed by a single π–π inter­action between stacked phen groups [centroid–centroid distance 3.5209 (11) Å], leading to a planar structure parallel to (010).

## Related literature

For related literature, see: Brown & Altermatt (1985 ▶); Janiak (2000 ▶); Harvey *et al.* (2006 ▶).
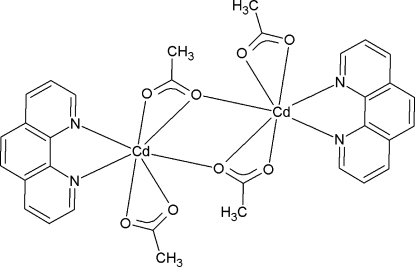


## Experimental

### 

#### Crystal data

[Cd_2_(C_2_H_3_O_2_)_4_(C_12_H_8_N_2_)_2_]
*M* *_r_* = 821.40
Orthorhombic, 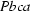

*a* = 8.4422 (7) Å
*b* = 15.6384 (13) Å
*c* = 22.2195 (18) Å
*V* = 2933.5 (4) Å^3^
*Z* = 4
Mo *K*α radiation
μ = 1.51 mm^−1^
*T* = 150 (2) K
0.50 × 0.40 × 0.20 mm

#### Data collection

Bruker SMART CCD area-detector diffractometer
Absorption correction: multi-scan (*SADABS*; Sheldrick, 2001 ▶) *T*_min_ = 0.50, *T*_max_ = 0.74
22676 measured reflections
3331 independent reflections
3062 reflections with *I* > 2σ(*I*)
*R*_int_ = 0.020

#### Refinement

*R*[*F* ^2^ > 2σ(*F* ^2^)] = 0.024
*wR*(*F* ^2^) = 0.064
*S* = 1.06
3331 reflections
210 parameters
H-atom parameters constrained
Δρ_max_ = 1.58 e Å^−3^
Δρ_min_ = −0.40 e Å^−3^

### 

Data collection: *SMART* (Bruker, 2001 ▶); cell refinement: *SAINT* (Bruker, 2001 ▶); data reduction: *SAINT*; program(s) used to solve structure: *SHELXS97* (Sheldrick, 2008 ▶); program(s) used to refine structure: *SHELXL97* (Sheldrick, 2008 ▶); molecular graphics: *SHELXTL* (Sheldrick, 2008 ▶); software used to prepare material for publication: *SHELXTL* and *PLATON* (Spek, 2003 ▶).

## Supplementary Material

Crystal structure: contains datablocks global, I. DOI: 10.1107/S1600536808029462/kj2097sup1.cif

Structure factors: contains datablocks I. DOI: 10.1107/S1600536808029462/kj2097Isup2.hkl

Additional supplementary materials:  crystallographic information; 3D view; checkCIF report

## Figures and Tables

**Table 1:** Selected bond lengths (Å)

Cd1—O13	2.2594 (15)
Cd1—O14	2.3239 (13)
Cd1—N1	2.3466 (15)
Cd1—N2	2.3890 (18)
Cd1—O14^i^	2.4398 (13)
Cd1—O24	2.4561 (15)
Cd1—O23	2.5425 (16)

Symmetry code: (i) 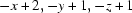.

**Fig. 1.**
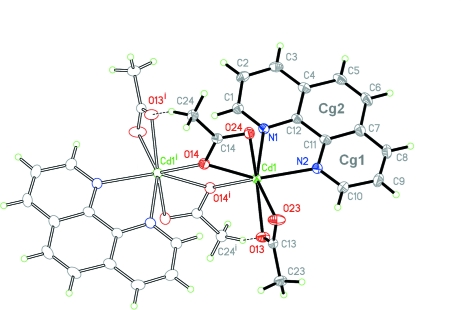
The dimeric unit of the title compound: the symmetry-independent part shown in full thermal ellipsoids, drawn at the 40% level. The intradimeric H-bond is shown in dashed lines.

**Fig. 2.**
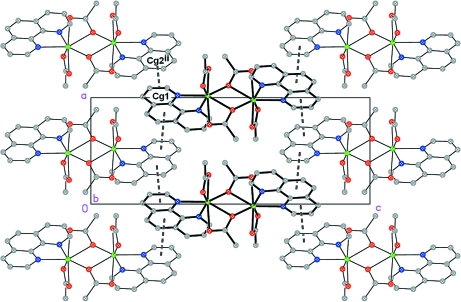
Packing view of the title compund down the [010] direction, showing the π···π bonded two-dimensional network.

## supplementary crystallographic information

### Comment

The title compound consists of dimeric units located around a crystallographic symmetry centre (Fig. 1) and made up of two Cd cations, two 1,10-phenanthroline (phen) molecules and four acetate anions. Each cadmium(II) unit is chelated by a phen group (through both nitrogen atoms), and two acetates (through their carboxylato oxygens). A seventh coordination bond adds to these three chelating bites, by way of one of the latter oxygens which acts also as a bridge linking both cadmium(II) centres (Fig. 1). Table 1 presents the coordination distances achieved. The Cd-Cd distance (Cd1···Cd1^i^: 3.846 (1)Å, (i): 2-x, 1-y, 1-z) as well as the O-Cd-O angle (O14-Cd1-O14^i^: 72.37 (5) °) are unexceptional.

This coordination scheme of the bis(µ_2_-acetato)-bis(acetato)-bis(L) type (L: a chelating aromatic amine) leading to a dimeric unit is not common among transition metal cations and in fact this is the first case reported.

The description of coordination geometries when chelating ligands are involved usually poses intrinsic difficulties which can be elegantly surmounted through a vectorial description of the ligand geometry, as proposed by Harvey *et al.* (2006) based on the Bond Valence Theory (Brown and Altermatt, 1985). When applied to the present case, the geometrical outcome turns out to be a tetrahedron, with angles between ligand vectors spanning the range 91.3 (1)–124.6 (1)°. The fact that the two main values associated with the theory, i.e. the bond valence sum (2.01 valence units, (v.u).) and the modulus of the bond valence vector (0.06 v.u.), agree almost perfectly with expected values (2.00 v.u. and 0.00 v.u., respectively) suggests a significant stability of this coordination sphere.

Regarding non-covalent interactions, there are just a few and not too strong either. The only hydrogen bond present (Table 2) is a non conventional, intramolecular one linking one of the methyl hydrogens to a carboxylato oxygen (Fig. 1). In fact the packing is governed by a single π···π interaction (Table 3 and Fig. 2) between staked phen groups, which gives raise to weakly interacting planar structures paralell to (010).

### Experimental

The title compound was obtained by direct mixing of two 0.15 M solutions of cadmium acetate dihydrate and 1,10-phenanthroline in dimethylformamide. Colorless needles began to develop at once, and after one day adequate crystals for X-ray diffraction could be extracted.

### Refinement

The hydrogen atoms were placed in geometrically idealized positions and allowed to ride on their parent atoms with C—H = 0.96–0.98Å and U_iso_(H) = 1.2/1.5× U_equiv_(C). Methyl groups were allowed to rotate around their 3-fold axis as well. A peak of ca. 1.5 eA^-3^ appears at 0.05 Å from Cd1. The next largest peak is less than 1.0 eA^-3^ in height.

### Crystal data

**Table:**

[Cd_2_(C_2_H_3_O_2_)_4_(C_12_H_8_N_2_)_2_]	*F*(000) = 1632
*M**_r_* = 821.40	*D*_x_ = 1.860 Mg m^−^^3^
Orthorhombic, *P**b**c**a*	Mo *K*α radiation, λ = 0.71073 Å
Hall symbol: -P 2ac 2ab	Cell parameters from 11288 reflections
*a* = 8.4422 (7) Å	θ = 2.7–27.8°
*b* = 15.6384 (13) Å	µ = 1.51 mm^−^^1^
*c* = 22.2195 (18) Å	*T* = 150 K
*V* = 2933.5 (4) Å^3^	Block, colourless
*Z* = 4	0.50 × 0.40 × 0.20 mm

### Data collection

**Table:**

Bruker SMART CCD area-detector diffractometer	3331 independent reflections
Radiation source: fine-focus sealed tube	3062 reflections with *I* > 2σ(*I*)
graphite	*R*_int_ = 0.020
φ and ω scans	θ_max_ = 27.9°, θ_min_ = 1.8°
Absorption correction: multi-scan (SADABS; Sheldrick, 2001)	*h* = −10→11
*T*_min_ = 0.50, *T*_max_ = 0.74	*k* = −19→20
22676 measured reflections	*l* = −28→27

### Refinement

**Table:**

Refinement on *F*^2^	Primary atom site location: structure-invariant direct methods
Least-squares matrix: full	Secondary atom site location: difference Fourier map
*R*[*F*^2^ > 2σ(*F*^2^)] = 0.024	Hydrogen site location: inferred from neighbouring sites
*wR*(*F*^2^) = 0.064	H-atom parameters constrained
*S* = 1.06	*w* = 1/[σ^2^(*F*_o_^2^) + (0.0401*P*)^2^ + 1.4472*P*] where *P* = (*F*_o_^2^ + 2*F*_c_^2^)/3
3331 reflections	(Δ/σ)_max_ = 0.003
210 parameters	Δρ_max_ = 1.58 e Å^−^^3^
0 restraints	Δρ_min_ = −0.40 e Å^−^^3^

### Special details

**Table:**

Geometry. All esds (except the esd in the dihedral angle between two l.s. planes) are estimated using the full covariance matrix. The cell esds are taken into account individually in the estimation of esds in distances, angles and torsion angles; correlations between esds in cell parameters are only used when they are defined by crystal symmetry. An approximate (isotropic) treatment of cell esds is used for estimating esds involving l.s. planes.

### Fractional atomic coordinates and isotropic or equivalent isotropic displacement parameters (Å^2^)

**Table:**

	*x*	*y*	*z*	*U*_iso_*/*U*_eq_	
Cd1	1.020491 (17)	0.537614 (8)	0.417981 (6)	0.01764 (7)	
N1	0.88137 (18)	0.41531 (9)	0.38716 (7)	0.0202 (3)	
N2	1.02282 (19)	0.52821 (10)	0.31067 (8)	0.0216 (4)	
C1	0.8037 (2)	0.36453 (13)	0.42461 (9)	0.0235 (4)	
H1	0.8129	0.3746	0.4666	0.028*	
C2	0.7084 (2)	0.29656 (12)	0.40510 (9)	0.0254 (4)	
H2	0.6519	0.2626	0.4333	0.030*	
C3	0.6983 (2)	0.28003 (12)	0.34466 (9)	0.0262 (4)	
H3	0.6364	0.2334	0.3305	0.031*	
C4	0.7800 (2)	0.33252 (12)	0.30367 (9)	0.0239 (4)	
C5	0.7738 (2)	0.32032 (14)	0.23972 (9)	0.0302 (4)	
H5	0.7160	0.2734	0.2238	0.036*	
C6	0.8487 (3)	0.37424 (14)	0.20153 (9)	0.0312 (5)	
H6	0.8441	0.3641	0.1594	0.037*	
C7	0.9354 (2)	0.44695 (13)	0.22400 (9)	0.0256 (4)	
C8	1.0118 (2)	0.50644 (16)	0.18593 (10)	0.0294 (5)	
H8	1.0089	0.4993	0.1435	0.035*	
C9	1.0896 (3)	0.57412 (14)	0.21075 (9)	0.0308 (4)	
H9	1.1416	0.6147	0.1858	0.037*	
C10	1.0919 (2)	0.58316 (13)	0.27331 (9)	0.0273 (4)	
H10	1.1455	0.6310	0.2900	0.033*	
C11	0.9455 (2)	0.46042 (11)	0.28630 (9)	0.0209 (4)	
C12	0.8686 (2)	0.40117 (11)	0.32713 (8)	0.0199 (4)	
C13	1.2479 (2)	0.66285 (12)	0.41072 (8)	0.0207 (4)	
C23	1.3877 (3)	0.72303 (14)	0.40927 (9)	0.0286 (4)	
H23A	1.3759	0.7629	0.3755	0.043*	
H23B	1.4855	0.6901	0.4043	0.043*	
H23C	1.3923	0.7552	0.4471	0.043*	
O13	1.27330 (18)	0.58473 (9)	0.42176 (6)	0.0282 (3)	
O23	1.11146 (17)	0.69086 (10)	0.40168 (8)	0.0354 (3)	
C14	0.7547 (2)	0.58393 (11)	0.48087 (8)	0.0192 (3)	
C24	0.6115 (2)	0.59980 (13)	0.51958 (9)	0.0252 (4)	
H24A	0.6170	0.6576	0.5365	0.038*	
H24B	0.6088	0.5579	0.5523	0.038*	
H24C	0.5155	0.5943	0.4951	0.038*	
O14	0.88527 (15)	0.56447 (8)	0.50710 (6)	0.0229 (3)	
O24	0.74741 (18)	0.59082 (9)	0.42510 (6)	0.0247 (3)	

### Atomic displacement parameters (Å^2^)

**Table:**

	*U*^11^	*U*^22^	*U*^33^	*U*^12^	*U*^13^	*U*^23^
Cd1	0.01769 (10)	0.01872 (10)	0.01652 (10)	−0.00151 (4)	−0.00046 (4)	−0.00019 (4)
N1	0.0207 (7)	0.0190 (7)	0.0210 (7)	0.0005 (6)	−0.0011 (6)	−0.0009 (6)
N2	0.0212 (9)	0.0241 (8)	0.0196 (8)	−0.0004 (6)	0.0005 (6)	−0.0009 (6)
C1	0.0231 (10)	0.0235 (10)	0.0238 (9)	0.0010 (8)	−0.0003 (7)	0.0000 (7)
C2	0.0223 (9)	0.0189 (9)	0.0350 (10)	−0.0002 (7)	0.0037 (8)	0.0016 (8)
C3	0.0209 (9)	0.0206 (9)	0.0372 (11)	−0.0006 (7)	−0.0017 (8)	−0.0047 (8)
C4	0.0205 (9)	0.0224 (9)	0.0290 (10)	0.0038 (7)	−0.0037 (7)	−0.0060 (7)
C5	0.0285 (10)	0.0310 (11)	0.0310 (10)	−0.0005 (8)	−0.0076 (8)	−0.0124 (9)
C6	0.0326 (11)	0.0398 (12)	0.0213 (9)	0.0053 (9)	−0.0055 (8)	−0.0101 (8)
C7	0.0231 (10)	0.0318 (10)	0.0220 (9)	0.0070 (8)	−0.0013 (8)	−0.0021 (8)
C8	0.0299 (11)	0.0401 (13)	0.0180 (9)	0.0071 (9)	0.0009 (7)	0.0013 (9)
C9	0.0321 (11)	0.0364 (12)	0.0240 (10)	0.0012 (9)	0.0056 (8)	0.0074 (8)
C10	0.0273 (10)	0.0292 (10)	0.0255 (10)	−0.0034 (8)	0.0028 (8)	0.0029 (8)
C11	0.0168 (9)	0.0213 (9)	0.0248 (10)	0.0044 (7)	−0.0034 (8)	−0.0024 (7)
C12	0.0169 (8)	0.0213 (9)	0.0217 (9)	0.0032 (7)	−0.0018 (7)	−0.0028 (7)
C13	0.0198 (9)	0.0245 (10)	0.0179 (8)	−0.0030 (8)	0.0003 (7)	−0.0008 (7)
C23	0.0239 (10)	0.0263 (10)	0.0355 (11)	−0.0050 (8)	−0.0032 (8)	0.0055 (8)
O13	0.0225 (7)	0.0234 (7)	0.0387 (8)	−0.0037 (6)	−0.0055 (6)	0.0065 (6)
O23	0.0200 (7)	0.0277 (8)	0.0584 (10)	0.0019 (6)	−0.0047 (7)	−0.0034 (7)
C14	0.0204 (8)	0.0138 (8)	0.0233 (9)	−0.0002 (6)	−0.0008 (7)	0.0005 (7)
C24	0.0228 (9)	0.0286 (10)	0.0243 (9)	0.0047 (8)	0.0014 (7)	0.0004 (8)
O14	0.0195 (6)	0.0261 (7)	0.0229 (6)	0.0029 (5)	−0.0011 (5)	0.0022 (5)
O24	0.0248 (7)	0.0291 (7)	0.0201 (6)	0.0023 (6)	−0.0005 (5)	0.0011 (5)

### Geometric parameters (Å, °)

**Table:**

Cd1—O13	2.2594 (15)	C6—H6	0.9500
Cd1—O14	2.3239 (13)	C7—C11	1.403 (3)
Cd1—N1	2.3466 (15)	C7—C8	1.413 (3)
Cd1—N2	2.3890 (18)	C8—C9	1.362 (3)
Cd1—O14^i^	2.4398 (13)	C8—H8	0.9500
Cd1—O24	2.4561 (15)	C9—C10	1.397 (3)
Cd1—O23	2.5425 (16)	C9—H9	0.9500
N1—C1	1.324 (2)	C10—H10	0.9500
N1—C12	1.356 (2)	C11—C12	1.450 (3)
N2—C10	1.329 (3)	C13—O23	1.248 (2)
N2—C11	1.358 (2)	C13—O13	1.265 (2)
C1—C2	1.402 (3)	C13—C23	1.510 (3)
C1—H1	0.9500	C23—H23A	0.9800
C2—C3	1.370 (3)	C23—H23B	0.9800
C2—H2	0.9500	C23—H23C	0.9800
C3—C4	1.407 (3)	C14—O24	1.246 (2)
C3—H3	0.9500	C14—O14	1.283 (2)
C4—C12	1.409 (3)	C14—C24	1.504 (2)
C4—C5	1.435 (3)	C24—H24A	0.9800
C5—C6	1.353 (3)	C24—H24B	0.9800
C5—H5	0.9500	C24—H24C	0.9800
C6—C7	1.442 (3)	O14—Cd1^i^	2.4398 (13)
			
O13—Cd1—O14	111.92 (5)	C8—C7—C6	122.93 (19)
O13—Cd1—N1	138.54 (5)	C9—C8—C7	119.28 (19)
O14—Cd1—N1	98.64 (5)	C9—C8—H8	120.4
O13—Cd1—N2	92.83 (5)	C7—C8—H8	120.4
O14—Cd1—N2	149.95 (5)	C8—C9—C10	119.21 (19)
N1—Cd1—N2	70.28 (5)	C8—C9—H9	120.4
O13—Cd1—O14^i^	83.11 (5)	C10—C9—H9	120.4
O14—Cd1—O14^i^	72.37 (5)	N2—C10—C9	123.3 (2)
N1—Cd1—O14^i^	80.16 (5)	N2—C10—H10	118.3
N2—Cd1—O14^i^	129.64 (5)	C9—C10—H10	118.3
O13—Cd1—O24	140.70 (6)	N2—C11—C7	122.70 (19)
O14—Cd1—O24	54.75 (4)	N2—C11—C12	117.68 (18)
N1—Cd1—O24	79.93 (5)	C7—C11—C12	119.61 (17)
N2—Cd1—O24	95.33 (5)	N1—C12—C4	122.03 (17)
O14^i^—Cd1—O24	118.94 (4)	N1—C12—C11	118.39 (16)
O13—Cd1—O23	54.03 (5)	C4—C12—C11	119.55 (17)
O14—Cd1—O23	95.70 (5)	O23—C13—O13	121.80 (18)
N1—Cd1—O23	151.38 (5)	O23—C13—C23	119.94 (18)
N2—Cd1—O23	85.03 (6)	O13—C13—C23	118.26 (18)
O14^i^—Cd1—O23	127.99 (5)	O23—C13—Cd1	67.36 (11)
O24—Cd1—O23	88.47 (5)	O13—C13—Cd1	54.44 (10)
C1—N1—C12	118.75 (16)	C23—C13—Cd1	172.67 (14)
C1—N1—Cd1	123.59 (13)	C13—C23—H23A	109.5
C12—N1—Cd1	117.36 (12)	C13—C23—H23B	109.5
C10—N2—C11	117.80 (18)	H23A—C23—H23B	109.5
C10—N2—Cd1	125.95 (13)	C13—C23—H23C	109.5
C11—N2—Cd1	116.25 (13)	H23A—C23—H23C	109.5
N1—C1—C2	123.00 (18)	H23B—C23—H23C	109.5
N1—C1—H1	118.5	C13—O13—Cd1	98.47 (12)
C2—C1—H1	118.5	C13—O23—Cd1	85.70 (12)
C3—C2—C1	118.82 (18)	O24—C14—O14	120.99 (17)
C3—C2—H2	120.6	O24—C14—C24	120.98 (17)
C1—C2—H2	120.6	O14—C14—C24	118.02 (16)
C2—C3—C4	119.59 (18)	O24—C14—Cd1	63.65 (10)
C2—C3—H3	120.2	O14—C14—Cd1	57.71 (9)
C4—C3—H3	120.2	C24—C14—Cd1	173.24 (13)
C3—C4—C12	117.74 (17)	C14—C24—H24A	109.5
C3—C4—C5	123.09 (18)	C14—C24—H24B	109.5
C12—C4—C5	119.15 (18)	H24A—C24—H24B	109.5
C6—C5—C4	121.43 (19)	C14—C24—H24C	109.5
C6—C5—H5	119.3	H24A—C24—H24C	109.5
C4—C5—H5	119.3	H24B—C24—H24C	109.5
C5—C6—C7	120.77 (18)	C14—O14—Cd1	94.46 (10)
C5—C6—H6	119.6	C14—O14—Cd1^i^	138.16 (11)
C7—C6—H6	119.6	Cd1—O14—Cd1^i^	107.63 (5)
C11—C7—C8	117.7 (2)	C14—O24—Cd1	89.32 (11)
C11—C7—C6	119.41 (19)		

Symmetry codes: (i) −*x*+2, −*y*+1, −*z*+1.

### Hydrogen-bond geometry (Å, °)

**Table:**

*D*—H···*A*	*D*—H	H···*A*	*D*···*A*	*D*—H···*A*
C24—H24B···O13^i^	0.98	2.51	3.313 (2)	139

Symmetry codes: (i) −*x*+2, −*y*+1, −*z*+1.

### Table 3 Table 3. π-π interactions (Å, °) for (I)

**Table:**

Group 1/Group 2	ccd	ipd	sa
Cg1/Cg2^ii^	3.5209 (11)	3.50 (1)	4(2)

Symmetry code: (ii)  1/2+x,y,1/2-zCg1: N2,C10,C9,C8,C7,C11 Cg2: C4,C5,C6,C7,C11,C12ccd: center-to-center distance (distance between ring centroids);  sa: mean slippage angle (angle subtended by the intercentroid vector to the plane normal); ipd: mean interplanar distance (distance from one  plane to the neighbouring centroid). For details, see Janiak (2000).

## References

[bb1] Allen, F. H. (2002). *Acta Cryst.* B**58**, 380–388. 10.1107/s0108768102003890 12037359

[bb2] Brown, I. D. & Altermatt, D. (1985). *Acta Cryst.* B**41**, 244–247.

[bb3] Bruker (2001). *SMART* and *SAINT* Bruker AXS Inc., Madison, Wisconsin, USA.

[bb4] Harvey, M. A., Baggio, S. & Baggio, R. (2006). *Acta Cryst.* B**62**, 1038–1042. 10.1107/S0108768106026553 17108658

[bb5] Janiak, C. (2000). *J. Chem. Soc. Dalton Trans.* pp. 3885–3898.

[bb6] Sheldrick, G. M. (2001). *SADABS* University of Göttingen, Germany.

[bb7] Sheldrick, G. M. (2008). *Acta Cryst.* A**64**, 112–122. 10.1107/S0108767307043930 18156677

[bb8] Spek, A. L. (2003). *J. Appl. Cryst.* **36**, 7–13.

